# Direct tumor visual feedback during free breathing in 0.35T MRgRT

**DOI:** 10.1002/acm2.13016

**Published:** 2020-09-15

**Authors:** Taeho Kim, Benjamin C. Lewis, Alex Price, Thomas Mazur, H. Michael Gach, Justin C. Park, Bin Cai, Erin Wittland, Lauren Henke, Hyun Kim, Sasa Mutic, Olga Green

**Affiliations:** ^1^ Department of Radiation Oncology Washington University School of Medicine St Louis MO 63110 USA; ^2^ Department of Radiology and Biomedical Engineering Washington University in St. Louis St Louis MO 63110 USA

**Keywords:** MR‐LINAC, MRgRT, visual guidance

## Abstract

To present a tumor motion control system during free breathing using direct tumor visual feedback to patients in 0.35 T magnetic resonance‐guided radiotherapy (MRgRT). We present direct tumor visualization to patients by projecting real‐time cine MR images on an MR‐compatible display system inside a 0.35 T MRgRT bore. The direct tumor visualization included anatomical images with a target contour and an auto‐segmented gating contour. In addition, a beam‐status sign was added for patient guidance. The feasibility was investigated with a six‐patient clinical evaluation of the system in terms of tumor motion range and beam‐on time. Seven patients without visual guidance were used for comparison. Positions of the tumor and the auto‐segmented gating contour from the cine MR images were used in probability analysis to evaluate tumor motion control. In addition, beam‐on time was recorded to assess the efficacy of the visual feedback system. The direct tumor visualization system was developed and implemented in our clinic. The target contour extended 3 mm outside of the gating contour for 33.6 ± 24.9% of the time without visual guidance, and 37.2 ± 26.4% of the time with visual guidance. The average maximum motion outside of the gating contour was 14.4 ± 11.1 mm without and 13.0 ± 7.9 mm with visual guidance. Beam‐on time as a percentage was 43.9 ± 15.3% without visual guidance, and 48.0 ± 21.2% with visual guidance, but was not significantly different (*P* = 0.34). We demonstrated the clinical feasibility and potential benefits of presenting direct tumor visual feedback to patients in MRgRT. The visual feedback allows patients to visualize and attempt to minimize tumor motion in free breathing. The proposed system and associated clinical workflow can be easily adapted for any type of MRgRT.

## 1 INTRODUCTION

In previous studies, tumors in the thorax were shown to move up to 5 cm^1^ and rotate up to 45°^2^ during respiration. Conventional respiratory motion‐compensation techniques such as surrogate‐based respiratory gating, breath‐hold, and marker‐based tumor tracking^3^, ^4^, ^5^ are clinically useful for tumor motion management but significant variations in cycle‐to‐cycle breathing can cause treatment inaccuracies.^6^, ^7^ Recently, several respiratory monitoring systems^3^, ^7^, ^8^, ^9^, ^10^ were introduced for respiratory motion management in radiotherapy providing respiratory guidance during radiotherapy in addition to medical imaging.^11^, ^12^ For instance, audio‐visual biofeedback^7^ uses a noninvasive external marker to measure abdominal motion and uses audio‐visual (AV) tools to return that information to the patient for respiratory motion guidance. Audio‐visual biofeedback can reduce average cycle‐to‐cycle variations in breathing displacement and period by up to 50% and 70%, respectively.^7^, ^13^ However, the applications of this system may be limited by an insufficient correlation between tumor and surrogate motion.^6^, ^14^

Real‐time 2D tumor tracking in MR‐guided radiotherapy (MRgRT) became clinically available in 2014.^15^, ^16^ MRgRT improves local control and spares critical organs by providing superior soft tissue contrast resolution and real‐time imaging‐based delivery. However, irregular tumor motion still hinders treatment efficiency in gated radiotherapy. Visual guidance systems in MRgRT have been introduced for improving tumor motion control in voluntary breath‐hold.^17^, ^18^ For example, Kim *et al*. displayed the treatment delivery system (TDS) operator screen inside the bore of the treatment system by using a video signal splitter and an MR‐compatible beam projector. In a similar approach, de Koste *et al*. displayed the TDS on an MR‐compatible monitor by using a video signal splitter and an adjustable mirror. The splitters supply the video signal of the TDS computer to an in‐room display device. There are two challenges associated with the splitter‐based approach: (a) the TDS display depends on video signal of a splitter; (b) displaying the entire TDS screen to patients includes unnecessary information that may confuse the patient, thus requiring further processing for advanced visual guidance.^18^

In our study, we developed a visual guidance system which does not impact the TDS display. In addition, a customizable visual guidance display was added without intensive programming that optimizes the information provided to the patient for efficient guidance. Through the study, we implemented the visual guidance system in a clinical workflow and investigated its impact on tumor motion control during free breathing in 0.35 T MRgRT.

## 2 MATERIALS AND METHODS

### 2.A 0.35T MR‐guided radiotherapy

**Fig. 1:**
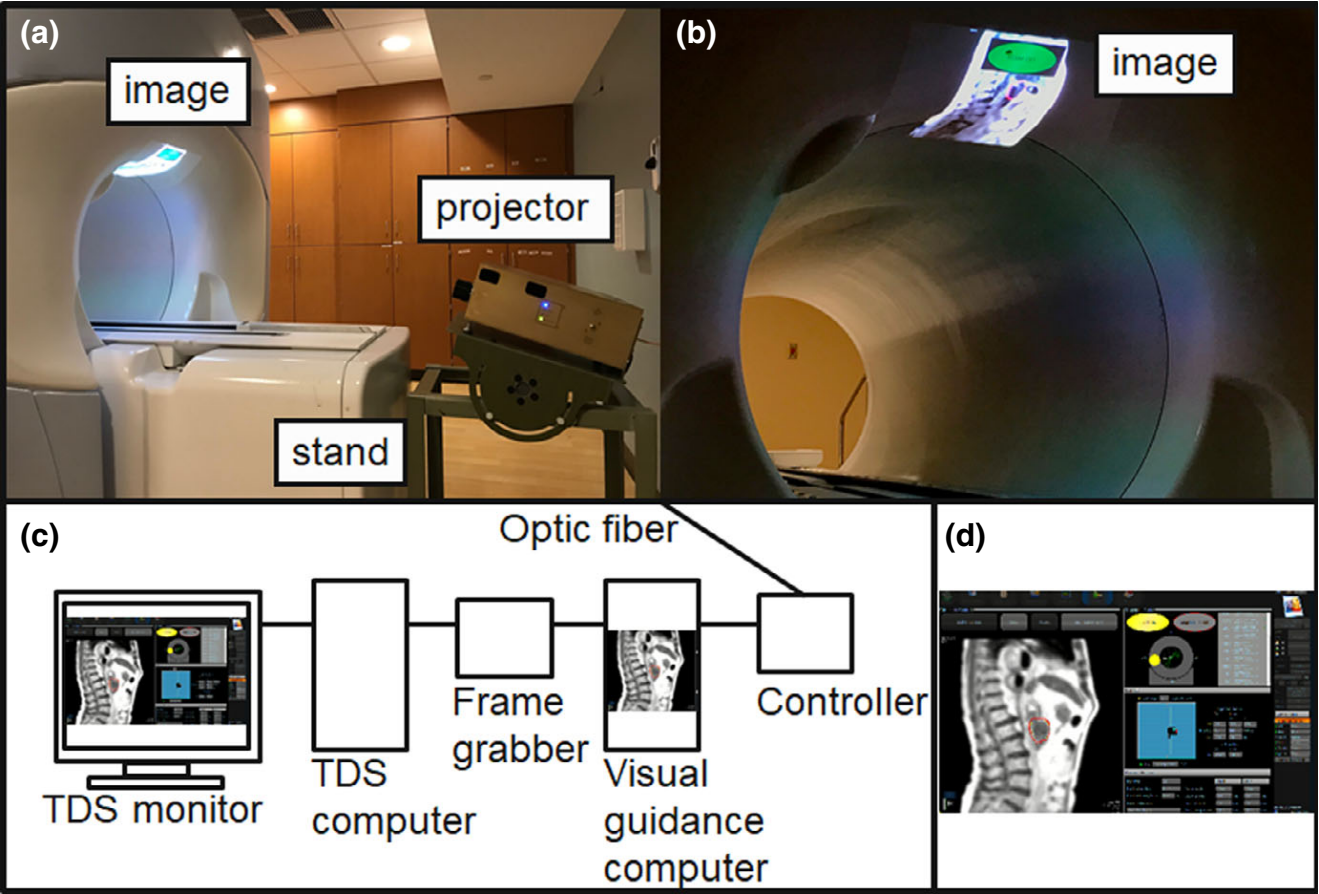
Direct tumor visualization system in 0.35 T MRgRT. (a) In‐room display system with magnetic resonance (MR)‐compatible projector and adjustable stand, (b) Image display inside the bore of the 0.35 T MRgRT treatment system. (c) Diagram of the video signal capturing, processing, and presentation system. (d) Example of the treatment delivery system display including cine magnetic resonance imaging and beam delivery information.

A 0.35 T MRgRT MRIdian system (ViewRay Inc., Oakwood Village, Ohio) with a linear accelerator radiation therapy delivery system was used in this study. In our institutional clinical workflow, volumetric MRIs are acquired using steady‐state precession (TrueFISP) in an axial orientation to position patients and localize treatment targets.^16^ After the clinical team had reviewed the MRIs, and verified the target contours and expected dose, 2D TrueFISP cine MRIs were acquired at 4 frames per second in a sagittal plane during MRgRT. The acquired spatial resolution was 0.35 × 0.35 cm^2^ with 5, 7, or 10 mm slice thicknesses, depending on the tracking efficiency. The tracking efficiency is related to the shape, contrast, and image texture of targets. Cine MRIs were displayed on the TDS monitor in real time during radiation delivery, as shown in Fig. 1(d). The TDS display included cine MRIs and beam delivery information. The system paused the cine MRI display during gantry motion due to significant moving metal‐related MRI artifacts.

### 2.B Direct tumor visualization system in 0.35T MRgRT

The direct tumor visualization system in the 0.35 T MRgRT environment is shown in Fig. 1. The direct tumor visualization system has three components, including: (a) video signal capture from the TDS system; (b) video signal processing for visual guidance; and (c) in‐room display.

First, we set up dual screen output on the TDS system with vendor's support such that the TDS display at the treatment console is independent from our signal capturing device. An Epiphan DVI2USB 3.0 screen grabber (Epiphan video, CA) was used in place of a video splitter to meet the high resolution (1900 × 1200) display requirements of the TDS for physician image viewing. The device was configured with the same resolution display extended display identification data (EDID) before connecting to the TDS system. Figure 1(c) shows how the frame grabber was connected between the TDS system and the visual guidance computer.

Second, Streamlabs open broadcast software (OBS), (Logitech International) was installed on the visual guidance computer to provide video signal capturing, processing, and presenting for visual guidance. Video signal processing using the software allowed personalization of the patient’s display. For example, we presented only 2D cine MRIs to the patient unlike previous studies that displayed the entire TDS display.^17^, ^18^ In addition, visual guidance‐specific notifications were added, such as a real‐time beam‐status indicator on the displayed images [Fig. 1(b)]. The processed video signal was sent to the in‐room projector through an optic fiber.

A Hyperion MRI digital projection system (Psychology Software Tools, Inc, PA) was used to convert and present the signal in the treatment room as shown in Fig. 1(a). As Kim *et al*. suggested, we displayed the images inside the bore of the treatment system. Additionally, an adjustable stand was developed and used to adjust the display location for patients and a projector keystone correction was used to minimize image distortion due to oblique projection as shown in Fig. 1(a).

### 2.C Clinical workflow of direct tumor visualization

**Fig. 2:**
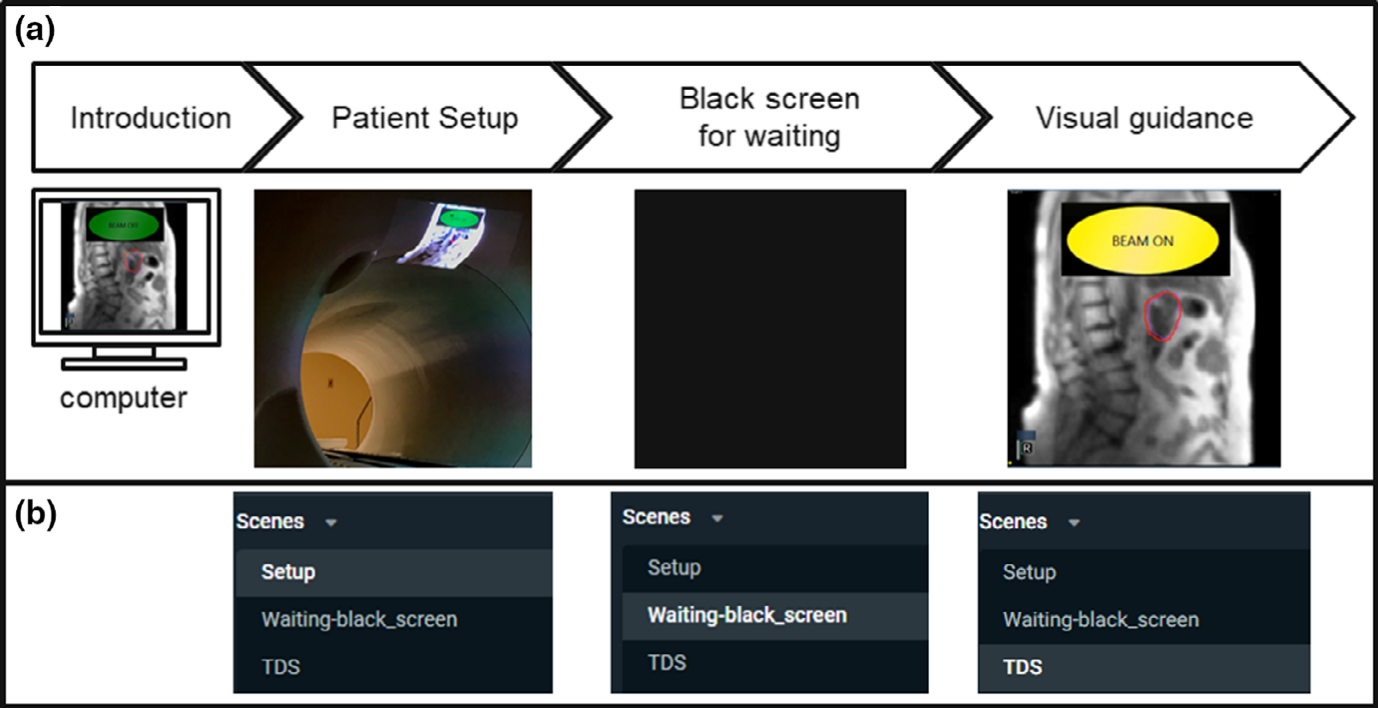
Clinical workflow of the direct tumor visualization. (a) Workflow diagram and corresponding example displays, and (b) Corresponding scene options on the live streaming software, Streamlabs open broadcast software.

Visual biofeedback based on direct tumor visualization was applied in our adaptive radiotherapy workflow.^15^, ^19^ The adaptive treatment planning and delivery workflows have been discussed in previous reports.^15^, ^16^ The additional workflow specific to providing direct tumor visualization to the patient is described below. Figure 2 presents an outline of the clinical workflow of the direct tumor visualization system, including: (a) introduction to patients; (b) patient setup; (c) black screen for waiting; and (d) visual guidance during treatment. First, before the patient enters the vault, they received a short introduction using a prepared demonstration set of cine MRIs displayed in the console area before the treatment started at the first fraction. The brief introduction included an overview of the MRIs, and descriptions of the target and auto‐segmented contours, and the beam‐status indicator displayed on the visual guidance. Orientative information about MRI images and abdominal anatomy was provided to patients since they may not have previously viewed human MRIs. Second, we projected the demonstration cine MRIs inside the bore of the treatment system while the patient was set up for treatment. The display location was adjusted using an adjustable stand based on patient position inside the bore. The size and focus of the image were adjusted based on the patient’s preferences. Patient‐specific adjustments were required due to differences in patient eyesight, immobilization position and location in the bore. Third, we projected a black screen during the treatment setup 3D MRI and the adaptive radiotherapy preparation process. Displaying the black screen reduced patient discomfort caused by the bright display and hid extraneous information from the patient that might confuse them or induce stress during the MRI and procedure preparation. Fourth, once beam delivery was ready to begin, we provided the visual guidance display to patients. As shown in Fig. 2, the visual guidance displayed only 2D cine MRIs with the target and auto‐segmented contours, and the beam‐status indicator. The size and position of the displayed information, including the beam‐status sign, were adjusted in real time while the patient was on the treatment table, if needed. Patients treated without visual guidance received the standard instructions to remain still and breathe normally throughout the treatment process and were given periodic reminders.

### 2.D Evaluation of tumor motion control and delivery time

Cine MRIs acquired during each treatment fraction were analyzed to evaluate tumor motion control with and without visual guidance. After treatment, the TDS produced a three channel RGB video file of the cine acquisition including gating contour and tumor tracking contours overlaid on the patient anatomy. In‐house software was developed in MATLAB 2019b (MathWorks) to isolate the contours from the underlying grayscale image. Once tracking contours were extracted from each serial MRI, they were combined to produce a probability distribution of tumor position. Tumor motion beyond the gating contour boundary in the inferior direction was quantified using superior‐inferior profiles. Only inferior motion was taken into consideration because the gating target placement within the boundary was at the full exhalation position, thus limiting superior excursion outside of the gating target. A significant‐difference analysis was performed using an unpaired *t*‐test. Tumor motion was evaluated for seven patients without visual guidance, for a total of 40 full or partial fractions (143 713 cine frames), and six patients with visual guidance, a total of 33 full or partial fractions (142 282 cine frames), including one patient treated both with and without visual guidance. This patient was treated with both methods due to changes in medical condition over the course of their treatment. Patients treated without visual guidance included two males and five females (age 55 to 71, average: 62.5), and patients treated with visual guidance included three males and three females (age 53 to 71, average: 61.7). The treatment locations without visual guidance include four pancreas, two liver, and one iliac. The treatment locations with visual guidance include three pancreas, one liver, one iliac, and one lymph node. The extent of motion and probability distribution were calculated. The treatment duty cycle was calculated as the percentage of frames which had the target contour entirely contained by the gating contour. Beam‐on time was recorded as a percentage, by the treatment machine, from when the beam‐on button was engaged to the end of radiation delivery, and included time required for gantry motion and when the beam was turned off for target gating (Beam‐on‐time/Treatment delivery time × 100). Duty cycle was calculated separately from beam‐on time by excluding time when the gantry was in motion.

## 3 RESULTS

### 3.A Implementation of direct tumor visualization

The direct tumor visualization system was successfully implemented on a 0.35 T MRgRT system in our clinic. Implementation included the system installation, training and instruction material preparation, workflow development, and staff training. System adjustment and optimization, updates to materials, and refresher training were conducted in a developing loop. Patient introduction to the system and projector adjustment required approximately seven minutes to perform prior to treatment, including five minutes for education at the first fraction and two minutes for projector adjustment at each fraction.

### 3.B Evaluation of tumor motion control and Beam‐on time

**Fig. 3:**
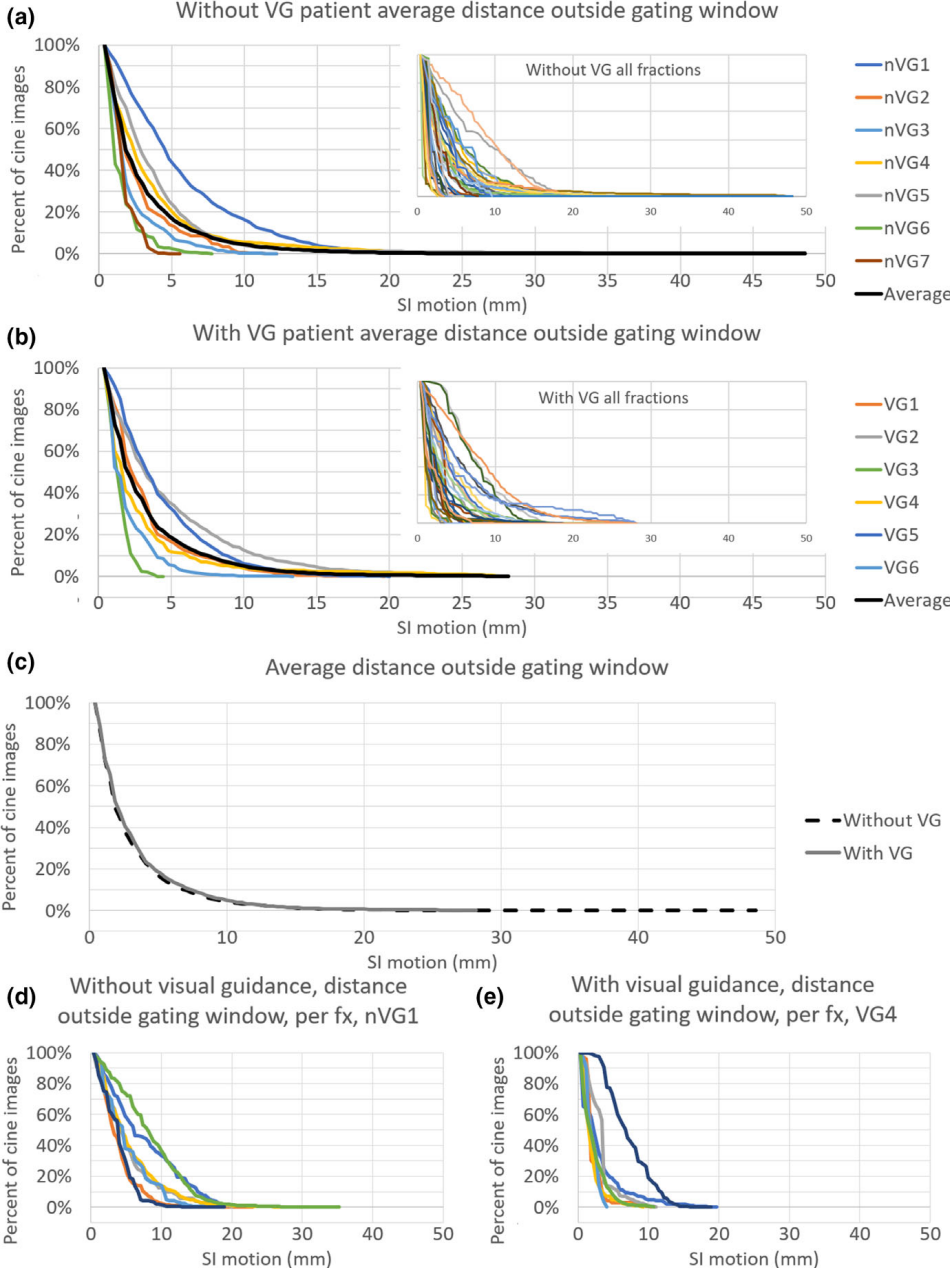
The percentage of cine magnetic resonance imaging (MRI) frames that the target tracking contour extends outside of the gating contour by the indicated distance shown as patient averages. The average of all patients is shown as a black line. (a) without visual guidance (nVG), and (b) with visual guidance (VG). (c) presents an average of all patients both without and with visual guidance (VG). (d) and (e) shows the target tracking contour motion outside of the gating contour for all individual fractions of a single patient without and with visual guidance, respectively. Both patients in (d) and (e) were treated for pancreatic disease.

**Fig. 4:**
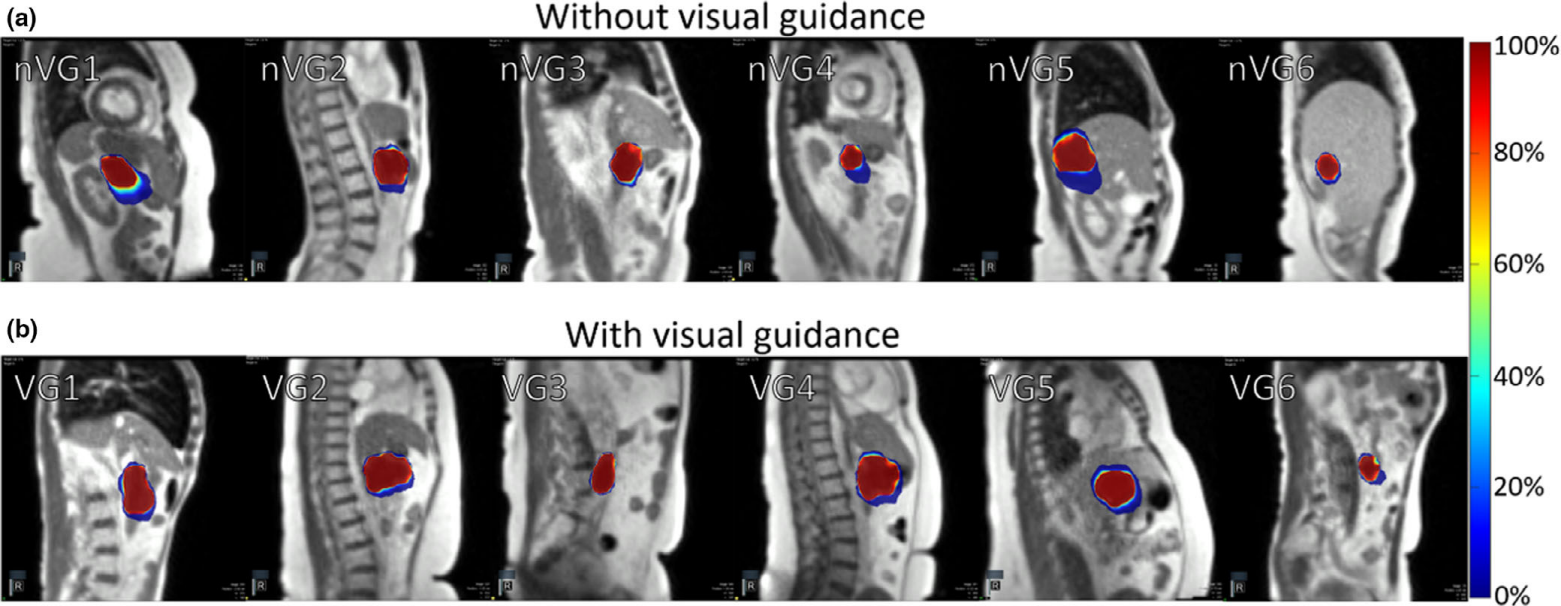
Distribution of auto‐segmented target contour from cine magnetic resonance imaging (MRI) without visual guidance (a), and with visual guidance (b) for a single fraction of six patients for each group. Individual patients are indicated by nVG(#) for no visual guidance and VG(#) with visual guidance in place. The color bar scale indicates the percent of frames that the region was encompassed by the auto‐segmented target contour.

**Fig. 5:**
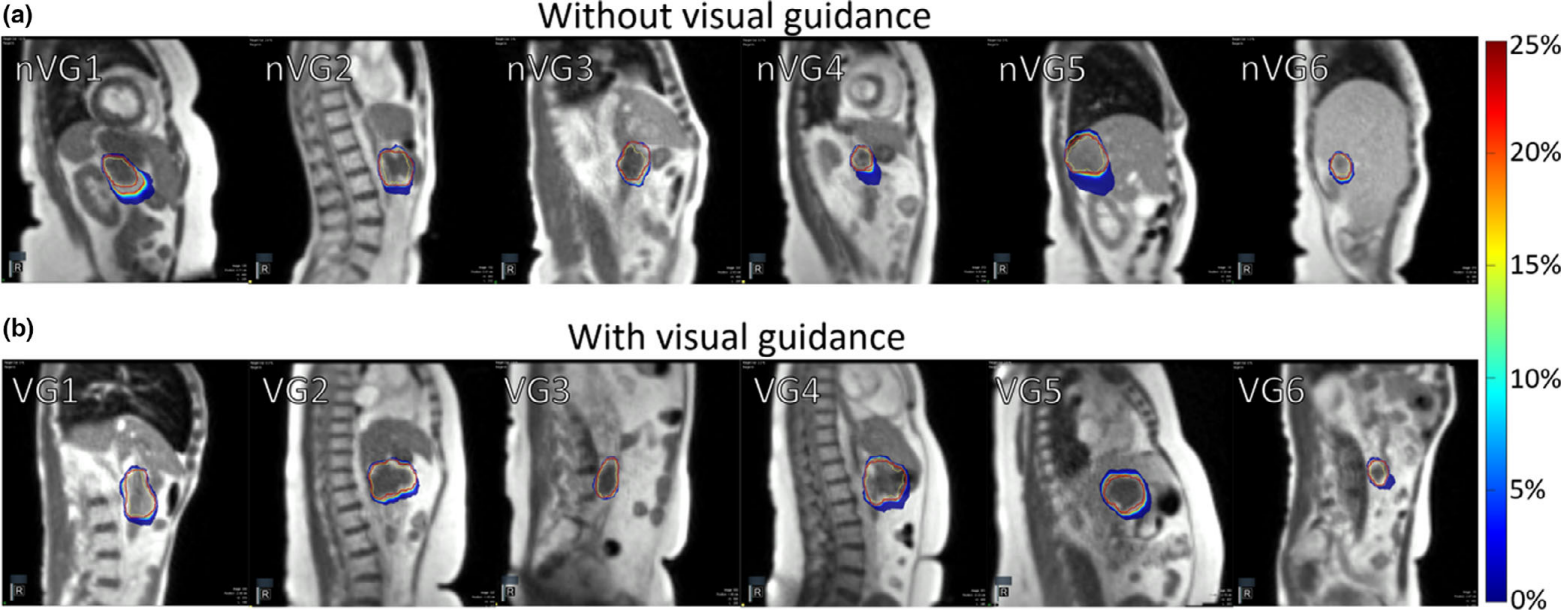
Distribution outliers from auto‐segmented target contour from cine magnetic resonance images (MRI) without visual guidance (a), and with visual guidance in place (b) for a single fraction of six patients for each group. Individual patients are indicated by nVG(#) for no visual guidance and VG(#) with visual guidance in place. The color bar scale indicates the percent of frames that the region was encompassed by the auto‐segmented target contour.

For the patients without visual guidance, the mean (±SD) of maximum tumor motion extent was 14.4 ± 11.2 mm (range, 2.2–48.2 mm). Patients with the visual guidance system in place had a mean (± SD) maximum tumor motion extent of 13.0 ± 7.9 mm (range, 3.3–27.8 mm). There was no significant difference in maximum tumor motion extent between the two groups (*P* = 0.54). When the target tracking contour was outside the gating contour, patients without visual guidance had an excursion of >1.9 mm for 50% of the imaging time and 2.0 mm for 50% of the imaging time with visual guidance in place. The target contour extended 3 mm outside of the gating contour (mean ± SD) for 33.6 ± 24.9% of the time without visual guidance, and 37.2 ± 26.4% of the time with visual guidance (*P* = 0.56). The patient treated both with and without visual guidance showed a maximum tumor motion extent of 5.2 and 4.1 mm without and with visual guidance in place, and the tracking contour was outside of the gating contour 53% of the imaging time both with and without visual guidance. The average distribution of motion extent for patients with and without visual guidance is shown in Fig. 3. The distribution of the auto‐segmented target tracking contour is shown in Fig. 4. The outlier motion, defined as occurring less than 25% of the cine MRI images, is shown in Fig. 5. The target tracking contour was completely inside the gating contour 74.5% ± 22.5% and 79.6% ± 25.5% of the time for patients without and with visual guidance, respectively, when imaging was being performed. There was no significant difference in the percentage of frames the target tracking contour was completely inside the gating contour (*P* = 0.63). Beam‐on time as a percentage had a mean (±SD) of 43.9 ± 15.3% without visual guidance, and 48.0 ± 21.2% with visual guidance. There was no significant difference in beam‐on time as a percentage between the two groups (*P* = 0.34). The mean (±SD) time from patient entry to exit of the treatment room was recorded by the radiation therapist and was 64.5 ± 22.7 min without visual guidance and 79.4 ± 14.8 min with visual guidance.

## 4 DISCUSSION

Direct tumor visualization for patient biofeedback in 0.35 T MRgRT was developed and implemented in our clinic. Compared to previous studies in MRgRT, the proposed system had multiple unique features including: (a) video signal capturing, (b) visual guidance projection, and (c) visual display layout.

In this study, we used a frame grabber instead of a video signal splitter. Since the TDS system requires a high display resolution, the TDS control room display resolution must not be compromised by any secondary display device. A secondary monitor or a projector might reduce the TDS display resolution, but the configured frame grabber does not. In addition, when a video signal splitter is powered off, neither output display receives a video signal because the video signal splitter produces both signals. In contrast, by using a frame grabber the TDS display remains independent and its operation is unaffected by screen grabber status.^17^, ^18^ This is important in preventing treatment delays due to potential splitter malfunction or troubleshooting.

Video signal capturing by the frame grabber provided more options in signal processing than using a video signal splitter. Once the video signal is received by the visual guidance computer, additional software can modify the acquired frames for generating customizable displays that can be altered in real time to patient or physician needs. For example, we used a live streaming freeware, Streamlabs OBS, to crop the original video signal to only display the cine MRI instead of the entire TDS screen, as shown in Fig. 1. We also added the beam‐status sign to assist patients in staying focused on motion control. Since the size and position of the main guidance display including the beam‐status sign can be adjusted in real‐time, patient‐specific adjustment can be applied. Additional display features for visual guidance and remote plan review, via live streaming, can be added using proper software, such as Streamlabs OBS. Although not specifically assessed by this study, showing only pertinent treatment aspects to the patient, instead of all the information including gantry angles and monitor units, may be important in reducing patient anxiety from excessive information.

A similar MRI‐compatible display was used in our study compared to previous studies.^18^ Since we displayed the images inside the bore of the treatment system using a projector like Kim *et al*., the resulting images were distorted due to the oblique projection, so a keystone function of the projector was used to correct image distortion. This approach is sufficient to remove the projection screen and adjustable mirror required for a conventional setup. In our approach, we developed an adjustable stand which can be used to adjust the projection angle and display location for patients compared to the approach of Kim *et al*. This system also does not require any additional hardware to be placed on the patient, eliminating the possibility of broken components or additional cleaning precautions.

Through this study, we investigated the implementation of the visual guidance system in a clinical workflow. The workflow included a brief introduction to the visual guidance system for patients. A demonstration video for the introduction and the patient setup was prepared. The demonstration video was especially helpful for educating patients who were unfamiliar with MRI, anatomical images, and the visual guidance process. We included a black screen option for use during waiting periods to prevent displaying unnecessary information to the patient, including 3D MR imaging and adaptive radiotherapy preparation procedures. In addition, it reduced patient discomfort from the bright background display of the projector. However, the black screen period between the setup and the visual guidance was long enough for patients to fall asleep so we informed the patients when the visual guidance started. Since cine MRIs were displayed on the TDS monitor once the treatment started, we ran the visual guidance session as the beam‐on button was pressed. It is noted that because the cine MRI displayed on the TDS system during gantry motion is not updated, the visual guidance appeared frozen to the patient.

Target tracking assessment of patients with and without the visual guidance system in place showed that the duty cycle remained similar. However, the target tracking contour was more likely to extend a greater distance outside of the gating contour without visual guidance. The target tracking contour had a greater range of motion outside the gating contour and was more likely to be outside of the target window, indicating that providing visual guidance has the potential benefit of reducing motion range. However, an additional study with randomized patient cohorts would be required to determine the true benefit. During treatment, patients were observed to be more conscious of their breathing and attempting to keep the displayed target contour within the gating contour, especially when the beam‐on indicator was active. Patients were not instructed to alter their breathing during the training session but changed their breathing pattern when presented with the beam‐on indicator. The addition of training time to familiarize patients with the system may reduce any total time benefit of the system, however time spent outside of the MRI bore is more desirable than treatment time in the bore where the patient may experience discomfort due to treatment positioning. It is noted that this study did not consider number of treatment beams, beam segments, monitor units, or setup difficulty, which could all have a significant impact on the total time spent in the treatment room by the patient. In the future, we will conduct additional studies to assess the time impact in further detail.

## 5 CONCLUSION

We demonstrated the clinical feasibility of direct tumor visualization to patients in MRgRT. It allows tumor motion control in free breathing, with the potential to reduce on‐table treatment time and tumor motion range. Clinical workflow for the proposed system can be easily adapted for any type of MRgRT.
